# Trends in intubation rates and durations in ventilated severely injured trauma patients: an analysis from the TraumaRegister DGU®

**DOI:** 10.1186/s13037-016-0109-6

**Published:** 2016-11-03

**Authors:** Khalid Almahmoud, Michel Teuben, Hagen Andruszkow, Klemens Horst, Rolf Lefering, Frank Hildebrand, Hans Christoph Pape, Roman Pfeifer

**Affiliations:** 1 Department of Orthopedics Trauma Surgery, University of Aachen Medical Center, 52074 Aachen, Germany; 2 Harald Tscherne Laboratory, RWTH Aachen University, 52074 Aachen, Germany; 3 Institute for Research in Operative Medicine (IFOM), University Witten/Herdecke, 51109 Cologne, Germany

**Keywords:** *Polytrauma*, *Intubation*, *Ventilation*, *Systemic Complications*, *Sepsis*, *MOF*

## Abstract

**Background:**

Endotracheal intubation in severely injured patients is known to be a risk factor for systemic complications. We aimed to examine the changes in intubation rates and durations in severely injured trauma patients, and rates of the systemic complications associated with ventilation changes by using a large trauma registry over the period of 13 years.

**Methods:**

Patient demographics, Injury Severity Score (ISS), ventilation days, ventilation free days (VFD), and prevalence of systemic complications (sepsis and multiple organ failure (MOF)) were obtained from the TraumaRegister DGU® and were compared over the study period.

**Results:**

During the study period (2002 – 2014), 35,232 patients were recorded in TraumaRegister DGU®. 72.7 % of patients (*n* = 25,629) were intubated, and 27.3 % (*n* = 9603) of patients did not require mechanical ventilation throughout their hospital stay. The mean age was 48 ± 21 years, mean ISS was 27.9 ± 11.5, mean length of ICU stay was 11.7 ± 13.8 days, mean time on mechanical ventilator was 7.1 ± 11.3 days, and mean ventilation free days (spontaneous respiration) was 19.5 ± 11.9 days. We observed a reduction in the intubation rates (87.5 % in 2002 versus 63.6 % in 2014), and early extubation (10 ventilation days in 2002, and 5.9 days in 2014) over time.

**Conclusion:**

Our study reveals a reduction in intubation rates and ventilation duration during the observation period. Moreover, we were able to observe decreased incidence of systemic complications such as sepsis over the 13 year study period, while no changes in incidence of MOF were registered. The exact relationship can not be proven in our study. This needs to be addressed in further analysis.

## Background

Mechanical ventilation is a criteria for intensive care unit admission, when there is a clinical sign that the patient cannot maintain or suitably protect their airway, or maintain adequate oxygenation or ventilation [1]. Studies have reported that intubation is a risk factor for ventilator-associated pneumonia and pulmonary infections [2]. In addition, pulmonary infections may trigger the development of systemic complications, such as acute respiratory distress syndrome (ARDS), sepsis or multiple organ failure (MOF) [3]. These serious clinical conditions affect the treatment strategies in trauma and may negatively influence the outcomes of trauma patients [4–6].

Factors that can affect pulmonary function include concurrent chest trauma and altered mental status [7, 10]. Recent studies have reported that 70–90 % of chest injuries are caused by blunt trauma [8]. The severity of pulmonary contusions correlates with the development of pulmonary infections, respiratory failure, and mortality [4, 6, 9]. Furthermore, severe impairment of consciousness due to a traumatic brain injury with a Glasgow Coma Score (GCS) ≤ 8 is considered to be an indication for intubation [10] according to the current guidelines [11]. Endotracheal intubation in trauma patient with a GCS ≤ 8 is also recommended in both pre-hospital and in-hospital settings [11].

The objective of this study was to test whether the intubation rates and duration of ventilation have changed over the last decade in severely injured trauma patients. Moreover, we aimed to examine whether changes in the incidence of systemic complications such as sepsis and multiple organ failure have occurred.

## Methods

### The trauma register DGU®

The TraumaRegister DGU® of the German Trauma Society (Deutsche Gesellschaft für Unfallchirurgie, DGU) was founded in 1993. The aim of this multi-centre database is to provide anonymous and standardized documentation of severely injured patients. Data are collected prospectively in four consecutive time phases from the site of the accident until discharge from hospital: A) pre-hospital phase, B) emergency room and initial surgery, C) Intensive Care Unit (ICU) and D) discharge. The documentation includes detailed information on demographics, injury patterns, comorbidities, pre- and in-hospital management, course of intensive care unit stay, relevant laboratory findings including data on transfusion, and outcome of each individual. The inclusion criterion is admission to hospital via the emergency room with subsequent ICU care.

The infrastructure for documentation, data management, and data analysis is provided by AUC-Academy for Trauma Surgery (AUC - Akademie der Unfallchirurgie GmbH), a company affiliated with the German Trauma Society. The scientific leadership is provided by the Committee on Emergency Medicine, Intensive Care and Trauma Management (Section NIS) of the German Trauma Society. The participating hospitals submit their data anonymously into a central database via a web-based application. Scientific data analysis is approved according to a peer review procedure established by Section NIS.

The participating hospitals are primarily located in Germany (90 %), but a rising number of hospitals in other countries contribute data as well (at the time of publication, this included Austria, Belgium, China, Finland, Luxembourg, Slovenia, Switzerland, The Netherlands, and the United Arab Emirates). Currently, approximately 25,000 cases from more than 600 hospitals are entered into the database per year.

The present study is in line with the publication guidelines of the TraumaRegister DGU® and registered as TR-DGU project ID 2011–024.

### Patient enrollment & clinical data collection

Data were obtained from the TraumaRegister DGU®. Patient’s records acquired between 1.4.2002 and 31.12.2014 were used. Patients eligible for enrollment in the study were at least 16 years of age, admitted to the ICU after being resuscitated, and had an Injury Severity Score (ISS) ≥ 16 points.

Clinical data, including age, gender, ISS, length of ICU stay (LOS), hospital LOS, days on mechanical ventilator, and mortality rates were collected. AIS-05 (according to the updated 2005 injury code) [12] and ISS scores were calculated for each patient [13, 14]. Laboratory results and other basic demographic data were recorded in the database.

### Study design & selection criteria

Patients’ clinical data were analyzed to assess the intubation rate, duration of ventilation and ventilator free days. The following criteria were used for patient selection: preclinical intubation and patient intubation during the clinical course in hospital, ventilator free days (VFD; defined as the number of days between successful weaning from mechanical ventilation and day 28 after study enrollment) (spontaneous respiration) [15], severe head and thoracic injuries as Abbreviated Injury Score (AIS) ≥ 3 [16]. The diagnosis of sepsis was made according to the criteria of the American College of Chest Physicians / Society of Critical Care Medicine (ACCP-SCCM) consensus conference committee [17, 18]. Sepsis was defined as the systemic response to infection, manifested by two or more of the following conditions as a result of infection: (1) temperature >38 °C or <36 °C; (2) heart rate >90 beats per minute; (3) respiratory rate >20 breaths per minute or PaC02 < 32 mm Hg; and white blood cell count > 12,OOOIcu mm, <4,OOO/cu mm, or > 10 % immature (band) forms.

Organ function status was evaluated according to the Sequential Organ Failure Assessment (SOFA) score [19]. Organ function was considered to be inappropriate and marked as organ failure when the SOFA score was ≥ 3. Moreover, multiple organ failure (MOF) was defined as simultaneous failure of at least two organs.

### Statistical analysis

Incidences are presented as percentages, while continuous values are presented as mean ± standard deviation (SD), and median (MD). Due to the large sample size, even minor differences between the groups tended to become statistically significant. Therefore formal statistical testing was largely avoided. The interpretation of results, however, focused on clinically relevant differences rather than on significant *P*-values. Data were analyzed using the Statistical Package for the Social Sciences (SPSS; Version 18, Chicago, IL, USA).

## Results

### Demographic data & clinical outcomes

Over the 13 year study period, 35,232 trauma patients admitted to the ICU were enrolled in the study. The majority of patients in the study population were male (73 %) with a mean age of 48 ± 2 years (MD 48 years), and a mean ISS of 27.9 ± 11.5 points (MD 25 points). The majority of patients had blunt injury (96 %). In this cohort, the average ICU LOS was 11.7 ± 13.8 days (MD 7 days), the mean number of days on a mechanical ventilator was 7.1 ± 11.3 days (MD 2 days), and the mean number of ventilator free days was 19.5 ± 11.9 days (MD 26 days) (Table 1). Accordingly, we identified 25,629 intubated patients and 9,603 not intubated patients (i.e., exhibited ventilation) throughout their hospital stay (Table 2).

**Table 1:** Demographics and outcomes of the study population presented as mean ± SD (SD = standard deviation)

	Study Participants *N* = 35232
Age (years) mean ± SD (median)	48 ± 21 (48)
Sex Ratio % (M:F)	73:27
Blunt: Penetrating Injury %	96:4
ISS (Points) mean ± SD (median)	27.9 ± 11.5 (25)
ICU LOS (Days) mean ± SD (median)	11.7 ± 13.8 (7.0)
Days on Mechanical Ventilation mean ± SD (median)	7.1 ± 11.3 (2.0)
Ventilation Free Days (VFD) mean ± SD (median)	19.5 ± 11.9 (26)
Hospital LOS (Days) mean ± SD (median)	24.7 ± 24.7 (19)
Mortality (%)	16.6

**Table 2:** Demographics and outcomes of the intubated sub-cohort, presented as mean ± SD (SD = standard deviation)

	Intubated Cohort *N* = 25629
Survivors: Non-Survivors ratio %	78.4 : 21.6
ISS (Points)	30.1 ± 12.1 (27)
Mechanical Ventilation Days	9.7 ± 12.2 (5)
Ventilation Free Days (VFD)	15.9 ± 11.8 (19)
Hospital LOS (Days)	27.2 ± 26.6 (21)

### Injury severity by body region in the intubated Sub-cohort

We sought to determine the severity and regions of injury in our intubated cohort. We observed a continuous decrease in the ISS (28.5 in 2002 and 26.8 in 2014), in GCS < 9 (34.4 % in 2002 and 28.3 % in 2014), and in rate of severe extremity injuries (AIS ≥ 3) (40.4 % in 2002 and 29.6 % in 2014) of the intubated cohort over the study period (2002–2014). However, we observed no differences in head injury (AIS-1 ≥ 3) (55.1 % in 2002 and 55.5 % in 2014) or chest injury patterns among patients (AIS-3 ≥ 3) (60.5 % in 2002 and 55.8 % in 2014) (Table 3).

**Table 3:** Mean Injury Severity Score (ISS) over the 13 year study period, and the associated components of head injury (AIS-1 ≥ 3), chest injury (AIS-3 ≥ 3), and extremity injury (AIS-5 ≥ 3). GCS (Glasgow Coma Scale)

Years	Head Injury (AIS-1 ≥ 3) %	Chest Injury (AIS-3 ≥ 3) %	Extremity Injury (AIS-5 ≥ 3) %	ISS Score (Points)	GCS < 9 %
2002	55.1	60.5	40.4	28.5	34.4
2003	56.3	54.3	37.9	28.6	34.0
2004	59.9	54.4	35.9	28.6	36.4
2005	57.0	53.9	35.8	28.2	33.4
2006	60.0	58.1	38.7	28.9	34.8
2007	57.9	58.1	39.0	29.0	31.3
2008	58.6	57.2	35.1	28.5	32.6
2009	54.5	59.2	37.5	28.3	32.8
2010	54.4	59.4	34.7	28.2	32.3
2011	54.7	59.7	33.6	27.9	29.9
2012	53.8	58.2	33.5	27.8	30.7
2013	55.6	54.7	30.3	27.1	28.8
2014	55.5	55.8	29.6	26.8	28.3

### Changes in ventilation rate and duration over time

**Fig. 1:**
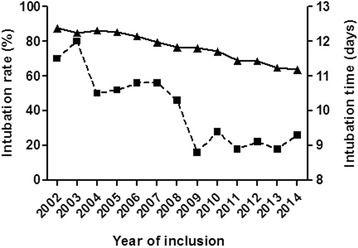
Changes in intubation rates and duration during the 13 year study period. Intubation rates showed a reduction over the course of the study. A pattern of earlier extubation was observed over time. *Legend: Intubation rates (triangles), Intubation duration (squares).* Intubation rate and duration over time

**Fig. 2:**
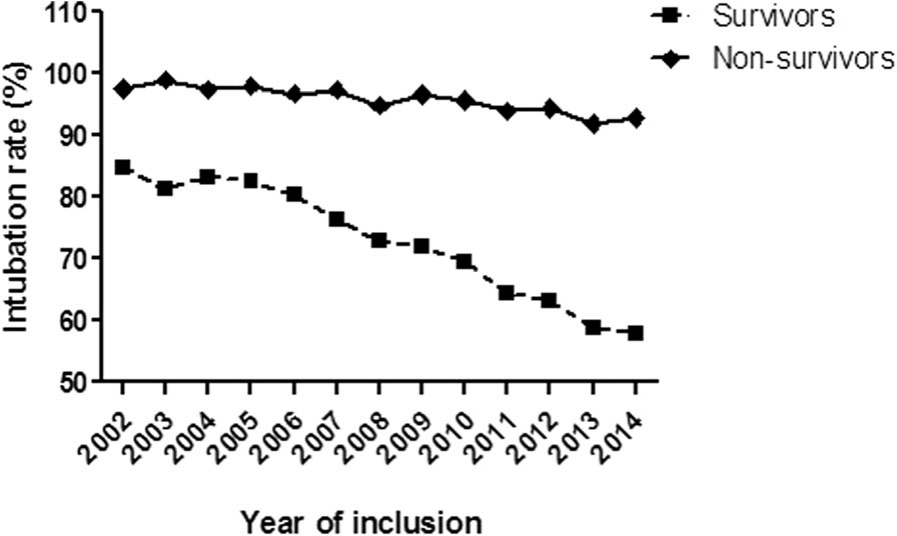
Changes in the intubation rates for survivors & non-survivors during the 13 year study period. We observed a reduction in the intubation rates for the survivors cohort when compared to the non-survivors. Intubation rate in survivors and non-survivors

During the study period, we observed a reduction in the intubation rates (87.5 % in 2002 versus 63.6 % in 2014) among all intubated patients (Fig. 1). We also observed earlier extubation at the beginning of the study period as compared to later, with an average of 10 ventilation days in 2002 and 5.9 days in 2014 (Fig. 1). Moreover, the intubation rates for the survivor’s cohort decreased when compared with the non-survivors (Fig. 2).

### Rate of systemic complications

**Fig. 3:**
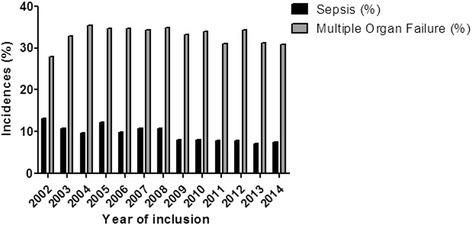
Systemic complications in the intubated cohort. No changes in the trends of MOF was observed. A minor continuous decrease in sepsis incidence was registered in the intubated cohort over time. Incidences of sepsis and multiple organ failure

We observed a minor decrease in the annual incidence of sepsis over the course of the study (13.1 % in 2002 and 7.5 % 2014). Moreover, there were no relevant changes in the incidence of multiple organ failure (28 % in 2002 and 30.9 % in 2014) over the study period (Fig. 3).

## Discussion

Over the past decade, improvements in the management of trauma patient care, such as transport to specialized trauma centers, early goal-directed therapy and resuscitation, have improved the outcome in severely injured patients. Moreover, ventilation in trauma patients is challenging because of the difficulty in achieving an optimal balance between sufficient ventilation and the avoidance of additional barotrauma to the lungs [20, 21].

We observed a marked decrease in the need for intubation over the study period. Accordingly, we observed a reduction in the intubation rates of the survivor’s cohort. This decrease might be associated with an overall decrease of the injury severity (ISS decrease over the years) and reduced rate of unconscious patients (GCS < 9). Especially, the incidence of severe (AIS ≥ 3) extremity injuries appear to has decreased over the observation period. One explanation is the continuous improvements of active and passive care safety and car safety regulations. However, the incidence of severe (AIS ≥ 3) chest and brain injuries did not change over the years. This gradual reduction in mechanical support may be explained by improvements in the treatment of severely injured patients as well [21, 22]. The latter changes have included consensus regarding the exact indications for intubation/ventilation, which have been introduced by surgical and anesthesiological societies (EAST, S3 Guideline). Moreover, the ATLS (Advanced Trauma Life Support) concept has become the standard for trauma care in emergency departments and advanced pre-clinical services and may also reduce the intubation rate due to a more standardized and strict approach. On the other hand, no major changes in the intubation rates for the non-survivors cohort were observed over the study period (97.6 % in 2002 and 92.9 % in 2014), which can be explained by the short observation period through the hospitalization, as the peak of deaths in most traumatic non-survivors patients is around the first 24 h post injury due to the direct head injury or massive bleeding.

With the increased recognition of the risks and economic consequences of prolonged ventilation, identification of strategies that reduce the duration of mechanical ventilation have been suggested [23–25]. Our data showed a reduction in the mechanical ventilator days over time, as in 2002 the mean number of mechanical ventilator days was 10 while the mean of 5.9 days was measured in 2014. Protocol–directed ventilator weaning has been shown to significantly reduce the duration of mechanical ventilation when compared with physician-directed methods [26, 27]. The likelihood of complications secondary to severe trauma is the consequence of the direct mechanical damage to the pulmonary parenchyma as well as the indirect systemic and pulmonary sequelae. Furthermore, the severity of pulmonary contusion correlates with the development of pulmonary infections, respiratory failure, sepsis, MOF, and mortality [4, 28]. Böhmer et al. have recently published an analysis of changes of ICU treatment over a period of 10 years [29] . This study confirmed a general trend towards reduced ventilation time and shorter stay in ICU as well. In comparison to our study, Böhmer et al. has included all patients (intubated and non-intubated) admitted to ICU also with less severe injuries (ISS ≥ 9). In addition, this analysis included only level 1 (supra-regional) and level 2 (regional) trauma care centers in Germany that participated continuously in the TR-DGU for 10 years (2002 to 2011). Thus the patient population considered by Böhmer et al. came from a much smaller group of hospitals.

Despite the changes in the ventilation rates and duration, we did not observe marked differences in the incidence of MOF and a minor decrease of sepsis incidence in our study; sepsis was reported in 11 % of patients in 2002, and 7.5 % in 2014 and the incidence of MOF was 28 % in 2002 and 30.9 % in 2014. The development of sepsis and MOF is dependent on multiple risk factors including the pattern of organ injury (abdominal, thoracic, head, etc.), presence of open fractures and soft tissue contamination, surgical strategy chosen (DCO (Damage Control Orthopedics) versus ETC. (Early Total Care)), and treatment in the ICU [6, 30–32]. These parameters could not be determined and evaluated in this study. It is very difficult to conclude that the decrease of the intubation reduced the rate of the systemic complications, since several factors beside the intubation, play a role. However, we feel that the intubation rate and short ventilation time might be indicators for improved treatment and decrease of the systemic complications.

We recognize that there are limitations to our study. TraumaRegister DGU® includes a large number of patients; their validity is restricted due to the detection of minor statistical differences without mandatory clinical relevance. In the absence of adequate statistical testing methods to manage this limitation we decided to present the descriptive variables of our study population without statistical significances. However, this does not restrict the identification of clinically significant effects and trends. Moreover, other parameters of interest, such as time point of intubation, Non-Invasive Ventilation (NIV), the presence of mis-intubation, number of re-intubation due to repeated surgery, lung function, lung injury, and development of ventilation associated pneumonia, might be of importance; however, these parameters are not registered in this database. Furthermore, no information was available regarding the indications for intubation, reintubation, and extubation in our cohort. Finally, we understand that our data registry contains data from several institutions that may use different practice guidelines and different methodology to determine the time for weaning.

## Conclusions

In conclusion, the current study demonstrates a decrease in the ventilation rates, and suggests an increase in early weaning over the last decade. Moreover, we have observed a decrease in the severity and patterns of injury over the study period. Importantly, we were able to observe decreased incidence of systemic complications such as sepsis over the 13 year study period, while no changes in incidence of MOF were registered. The exact relationship can not be proven in our study. This needs to be addressed in further analysis.

## Abbreviations

AIS: Abbreviated Injury Score
ICU: Intensive Care Unit
ISS: Injury Severity Score
MODS: Multiple Organ Dysfunction Score
MOF: Multiple organ failure
SIRS: Systemic Inflammatory Response Syndrome
Total LOS: Total Length of Stay
VFD: Ventilator free days
